# Thermophilic Nucleic Acid Polymerases and Their Application in Xenobiology

**DOI:** 10.3390/ijms232314969

**Published:** 2022-11-29

**Authors:** Guangyuan Wang, Yuhui Du, Xingyun Ma, Fangkai Ye, Yanjia Qin, Yangming Wang, Yuming Xiang, Rui Tao, Tingjian Chen

**Affiliations:** MOE International Joint Research Laboratory on Synthetic Biology and Medicines, School of Biology and Biological Engineering, South China University of Technology, Guangzhou 510006, China

**Keywords:** thermophilic organisms, thermophilic enzymes, nucleic acid polymerases, xeno nucleic acids (XNAs), xenobiology

## Abstract

Thermophilic nucleic acid polymerases, isolated from organisms that thrive in extremely hot environments, possess great DNA/RNA synthesis activities under high temperatures. These enzymes play indispensable roles in central life activities involved in DNA replication and repair, as well as RNA transcription, and have already been widely used in bioengineering, biotechnology, and biomedicine. Xeno nucleic acids (XNAs), which are analogs of DNA/RNA with unnatural moieties, have been developed as new carriers of genetic information in the past decades, which contributed to the fast development of a field called xenobiology. The broad application of these XNA molecules in the production of novel drugs, materials, and catalysts greatly relies on the capability of enzymatic synthesis, reverse transcription, and amplification of them, which have been partially achieved with natural or artificially tailored thermophilic nucleic acid polymerases. In this review, we first systematically summarize representative thermophilic and hyperthermophilic polymerases that have been extensively studied and utilized, followed by the introduction of methods and approaches in the engineering of these polymerases for the efficient synthesis, reverse transcription, and amplification of XNAs. The application of XNAs facilitated by these polymerases and their mutants is then discussed. In the end, a perspective for the future direction of further development and application of unnatural nucleic acid polymerases is provided.

## 1. Introduction

Nucleic acid polymerases are enzymes that catalyze DNA or RNA synthesis, including DNA polymerases (DNAPs), RNA polymerases (RNAPs), reverse transcriptases (RTs), and RNA-dependent RNA polymerases (RdRps), which play central roles in the storage and transmission of genetic information in living organisms, and have been widely applied in molecular biology and biotechnology [1,2,3,4,5]. Their unique activities and functions have laid the foundation of many broadly used or modern techniques, including polymerase chain reaction (PCR), DNA sequencing, and DNA information storage [6,7,8,9]. Thermostability is a desired property of nucleic acid polymerases for many of their applications, especially those involving thermocycling. Therefore, nucleic acid polymerases derived from thermophilic microorganisms have been widely used in biotechnology, due to their innate tolerance towards high temperatures [10,11], and some of them have been further engineered to be hyperthermophilic for better performance in these applications [12].

Due to the limited number of building blocks, natural DNA and RNA have intrinsic constraints for their properties, functions, and applications, which in principle, can be expanded by incorporating more kinds of building blocks. The past few decades have witnessed the development of xeno nucleic acids (XNAs). The attempts to create these DNA and RNA analogs with unnatural moieties have fostered a new field named xenobiology. The term “xenobiology” derives from the Greek word *xenos*, which means “foreign, alien, or stranger” [13]. The main aim of xenobiology is to expand the framework of natural life forms with artificial building blocks, such as XNAs and non-canonical amino acids (ncAAs). These building blocks are orthogonal with natural components, which leads to increased genetic and functional diversity [14]. In addition, the new-to-nature feature of xenobiology prevents information exchange with natural systems, forming a “genetic firewall” [15].

Similar to DNA and RNA, XNAs also need to be enzymatically manipulated for broader application. For example, enzymatic synthesis and amplification of XNAs are essential for efficiently producing and evolving functional XNA molecules [16]. However, due to their exotic structures, the unnatural nucleoside triphosphates of many XNAs are poor substrates for natural nucleic acid polymerases, and thus employing protein engineering approaches to make efficient XNA polymerases (XNAPs) is one of the most urgent tasks in xenobiology, which has drawn broad research interest in recent years [17,18,19,20]. Many of the thermophilic nucleic acid polymerases have been used as the starting scaffolds for generating XNAPs, and their great thermostability is a useful feature for high-temperature synthesis and thermocycling amplification of XNAs [21]. With the engineered XNAPs, the great potential of XNAs in a broad range of applications has been extensively demonstrated [22,23].

In this review, we first summarize the thermophilic and hyperthermophilic nucleic acid polymerases that have been extensively studied and broadly applied in biotechnology. The strategies for engineering these polymerases to be efficient XNAPs are introduced next, followed by the summary of representative XNAPs, with the relationship of key mutations and activities discussed. The applications of XNAPs are then reviewed.

## 2. Thermophilic and Hyperthermophilic Nucleic Acid Polymerases

**Figure 1:**
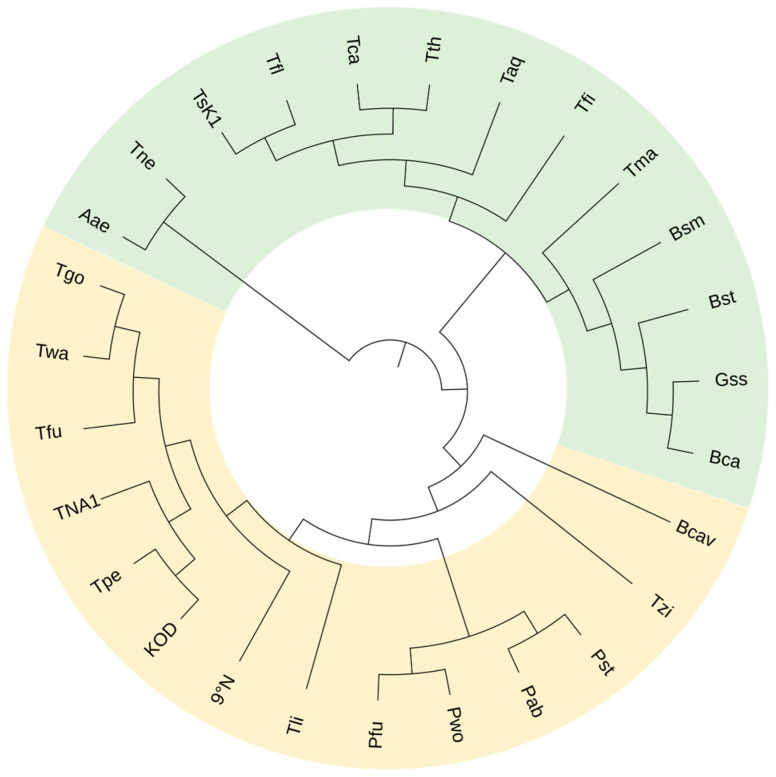
Phylogenetic tree of representative thermophilic DNAPs. Green: family A DNAPs; pale yellow: family B DNAPs.

Generally speaking, extensively investigated and applied thermophilic and hyperthermophilic nucleic acid polymerases mainly include thermostable DNAPs and minority RNAPs mostly from *Thermus, Thermococcus*, *and Pyrococcus* [5,10,24] (Table 1). From the perspective of family classifications, the DNAPs among these enzymes are concentrated in family A and B (Figure 1).

Taq DNAP from the thermophilic bacterium *Thermus aquaticus* was the first isolated thermophilic DNAP [54], which led to a breakthrough in PCR technology by eliminating the addition of a new enzyme after each cycle of thermocycling [55,56]. The optimal temperature of Taq DNAP is 75 to 80 °C, which is much higher compared with DNAPs from organisms living in regular environments. However, its half-lives are 45 to 50 min at 95 °C and 9 min at 97.5 °C, which are relatively short [26]. Taq DNAP has been classified as family A. It has 5′-3′ exonuclease activity and no 3′-5′ exonuclease activity, so its fidelity is not good compared to polymerases that own 3′-5′ exonuclease activity [57]. Under optimized conditions, the error rate of Taq DNAP was tested to be about 1.2 × 10^−5^ to 3.3 × 10^−6^ mf × bp^−1^ × d^−1^ (mutation frequency per base pair per duplication) [25]. Based on the real-time PCR experiments, amplification efficiencies of Taq DNAP were found to be around 80% for targets shorter than 1 kb and around 60% for 2.6 kb targets with a CG content between 45 to 56% [58]. Efforts have been made to alter the properties of Taq DNAP to improve its performance for different applications. For example, the pH of the reaction buffer and MgCl_2_ concentration have been optimized to improve its fidelity [25]. Deletion of proper regions in the 5′-3′ exonuclease domain has proven effective in improving the fidelity or thermostability of Taq DNAP. KlenTaq, a truncated variant of Taq DNAP lacking the N-terminal 235 amino acids, has been reported to have a two-fold higher fidelity than that of Taq DNAP [59]. A similar variant, the Stoffel fragment (SF), which is deficient in the N-terminal 289 amino acids, was found to have an increased thermostability [26]. Besides the DNA amplification activity, Taq DNAP also demonstrated some extent of RNA RT activity [60]. With optimized conditions, Taq DNAP could facilitate one-enzyme reverse transcription-qPCR of viral RNA [61].

Similar A-family thermophilic DNAPs have been identified from other *Thermus* strains, such as Tfi DNAP from *Thermus filiformis* [62], Tth DNAP from *Thermus thermophilus* [28], Tfl DNAP from *Thermus flavus* [63], Tca DNAP from *Thermus caldophilus* [30], and TsK1 DNAP from *Thermus scotoductus* [31]. Like Taq DNAP, these DNAPs possess 5′-3′ exonuclease activity but no 3′-5′ exonuclease activity. The PCR performances, such as amplification efficiency, fidelity, and specificity, and reaction conditions of Tfi DNAP, are similar to those of Taq DNAP [27]. Removing the 5′-3′ exonuclease domain of Tfi DNAP did not significantly affect the enzyme activity and stability [64]. However, a comparative study of exo^–^/exo^+^ Tfi DNAP blends with different blending ratios exhibited that raising the proportion of exo^–^ Tfi mutant led to an increase in the PCR amplification yield for the target product [65]. Tth DNAP from *Thermus thermophilus* HB8 also showed structural and functional similarities with Taq DNAP [66]. In addition, under similar conditions in the presence of Mn^2+^, Tth DNAP performed higher reverse transcription activity than Taq DNAP, which is useful for one-pot reverse transcription and PCR amplification of low-level RNA [67]. Sso7d is a small protein isolated from hyperthermophilic archaebacteria *Saccharolobus solfataricus* and may play a role of stabilizing the genomic DNA in the cell [68,69]. It has great thermostability and is able to bind with dsDNAs without much sequence preference [70]. An early study found that the fusion of this protein with several DNAPs significantly enhanced their processivity [71]. Recently, Sso7d protein was also fused to the N-terminal of Tth DNAP, which might improve the DNA binding capacity and processivity of Tth DNAP without affecting its catalytic activity and stability [66].

Though with a high sequence homology with Taq DNAP, some family A DNAPs from other *Thermus* strains exhibit some different characteristics. For example, Tfl DNAP from *Thermus flavus* demonstrated a higher thermostability and maintained PCR activity after heat treatment at 94 °C for 60 min, while Taq DNAP lost activity within 30 min under the same temperature [29]. Furthermore, Tfl and Tth DNAPs can significantly eliminate negative influences from the inhibitors of PCR reaction in the intraocular fluids and blood, avoiding false-negative results [72,73]. For another example, Tca DNAP exhibited longer half-lives in the presence of gelatin and a narrower working pH range than that of Taq DNAP [30,74]. Recently, a novel A-family DNAP, TsK1 DNAP, was reported to have a comparable half-life to rTaq (a commercially available recombinant Taq DNAP), which is shorter than that of Taq DNAP [31]. However, this enzyme demonstrated high amplification efficiency and better fidelity than Taq DNAP, making it a potential tool for molecular biology methodologies.

Many other family A DNAPs have been isolated from *Bacillus* species [75], such as Bst DNAP from *Bacillus stearothermophilus* [76] (now categorized as *Geobacillus stearothermophilus* [77]), Bca DNAP from *Bacillus caldotenax* [33], Bcav DNAP from *Bacillus caldovelox* [34], Bsm DNAP from *Bacillus smithii* [78], and Gss DNAP from *Geobacillus* sp. 777 [36]. The optimal temperatures of these DNAPs are 60 to 70 °C, which are lower than those of the thermostable polymerases from *Thermus* species introduced above. Bst-like DNAPs are widely used in isothermal amplification techniques, such as loop-mediated isothermal amplification (LAMP) and whole genome amplification (WGA), due to their strong strand displacement activity [79,80].

Hyperthermophilic microorganisms are bacteria or archaea whose optimal temperature for growth exceeds 80 °C [81]. *Thermotoga*, *Thermosipho*, *Aquifex*, and *Thermocrinis* are common genera of hyperthermophilic bacteria [82]. Tma DNAP isolated from *Thermotoga maritima* is a 97 kDa A-family polymerase with inherent 3′-5′ proofreading activity and 5′-3′ exonuclease activity [37]. Tma DNAP exhibited activity over a wide range of temperatures from 45 to 90 °C, with the optimal temperature being 75 to 80 °C. N-terminal truncation of Tma DNAP yielded UlTma (Perkin-Elmer) with enhanced thermostability [83]. The presence of 3′-5′ proofreading activity does not confer a high level of fidelity to UlTma, as implied by a similar replication accuracy with that of Taq DNAP [84]. A similar polymerase, Tne DNAP, has been isolated from *Thermotoga neapolitana* [38]. Later research found that mutations in the O-helix region improved the fidelity of this polymerase [39]. A mixture of KlenTaq and Tne DNAPs has also been prepared and found useful for the efficient amplification of long DNA fragments [85]. Aae DNAP isolated from *Aquifex aeolicus* is another family A DNAP, possessing 5′-3′ polymerase activity and 3′-5′ proofreading activity but no 5′-3′ exonuclease activity [40]. Half-lives of Aae DNAP, in the presence of BSA, were 6 h and 1.7 h at 75 and 85 °C, respectively. Although *Aquifex aeolicus* can grow at nearly 95 °C, the activity of Aae DNAP decreased rapidly at temperatures over 90 °C.

Family B DNAPs from hyperthermophilic archaea have been widely used in PCR due to their good thermostability and 3′-5′ proofreading activity [42,86]. Several thermostable DNAPs have been isolated from the genera *Thermococcus* and *Pyrococcus*, characterized, and commercialized. Tli (Vent) DNAP from *Thermococcus litoralis* is an archaeal DNAP, with a molecular weight of 89 kDa [41]. It is also the first reported thermostable DNAP possessing proofreading activity, which demonstrated a 2–4 times lower error rate compared to the proofreading activity-free enzyme Replinase DNAP (isolated from *Thermus flavis*) [41]. Tli DNAP is extremely thermostable, having a half-life of 2 h at 100 °C, and can be used for high-temperature DNA synthesis. In addition, Tli DNAP is resistant to hemoglobin inhibition, making it suitable for PCR amplification of DNAs in blood samples [72]. KOD DNAP is another commercial high-fidelity B-family polymerase possessing a 3′-5′ exonuclease domain and was isolated from *Thermococcus kodakaraensis* [42] (formerly *Pyrococcus* sp. KOD1 [87]). KOD DNAP has a higher thermostability than most DNAPs, and its half-life at 95 °C reaches 12 h. It has also been reported to have an extension rate of 6.0–7.8 kb/min and an error rate of 2.6 × 10^−6^, allowing efficient and faithful amplification of DNA in PCR reaction. PCR technique based on KOD DNAP was further developed for accurate amplification of long DNAs [88]. With a mixture of wild-type KOD DNAP and its exo^–^ variant (N210D), i.e., KOD Dash polymerase, long DNA fragments (up to 15 kb) were accurately amplified. 9°N DNAP, isolated from *Thermococcus* sp. 9°N-7, has a similar temperature-sensitive strand displacement activity and K_m_ values with Tli DNAP [43]. Tgo DNAP, isolated from *Thermococcus gorganarius*, is another widely engineered polymerase for XNA synthesis [44]. Besides these DNAPs introduced above, many other B-family polymerases have also been isolated from *Thermococcus* species and characterized, including Tfu DNAP from *Thermococcus fumicolans* [45], TNA1 DNAP from *Thermococcus* sp. NA1 [46], Tpe DNAP from *Thermococcus peptonophilus* [47], Tzi DNAP from *Thermococcus zilligii* [48], and Twa DNAP from *Thermococcus waiotapuensis* [49].

Pfu DNAP is one of the most representative family B DNAPs isolated from hyperthermophilic marine archaea *Pyrococcus furiosus* [86]. Pfu DNAP has a high fidelity under optimized buffer and substrate concentrations [50]. The error rate of Pfu DNAP was found to be 1.38 × 10^−6^. This high fidelity is mainly attributed to the 3′-5′ exonuclease activity, and an exo^–^ mutant of Pfu DNAP demonstrated significantly decreased fidelity [50]. The extension rate of Pfu DNAP is only 0.5–1.5 kb/min, which is lower than that of most other DNAPs [10]. The fusion of the Sso7d protein to the N-terminal of Pfu DNAP improved its processivity but did not affect its catalytic activity and stability [71]. It was also found that Pfu DNAP has weak incorporation activity of dUTP, which reduced the PCR efficiency when dUTP was used in PCR reaction [89].

Pst DNAP, also known as Deep Vent DNAP, is another well-studied thermophilic archaeal DNAP. It was isolated from *Pyrococcus* strain GB-D, which can grow at 104 °C [90]. Pst DNAP possesses a high fidelity with an error rate of 2.7 × 10^−6^, better than that of Vent or Taq DNAP [50]. Like other B-family polymerases, Pst DNAP has a high 3′-5′ proofreading activity that decreases errors during the DNA replication process. Deletion of the 3′-5′ exonuclease activity also significantly reduced its fidelity [51].

Several other thermophilic DNAPs that are not as famous as Pfu and Deep Vent DNAPs have been isolated from other *Pyrococcus* species. For example, Pab DNAP, which is also a B-family DNAP, was isolated from *Pyrococcus abyssi*, an archaeon growing in hyperthermal environments in the deep sea [91]. Pab DNAP has a higher thermostability than Taq and Pfu DNAPs and retains 75% of its activity after being incubated at 100 °C for 5 h [52]. Pab DNAP also has 3′-5′ exonuclease activity that confers proofreading ability and high fidelity to it [52]. Another example is Pwo DNAP from *Pyrococcus woesei* [92]. This DNAP has a molecular weight of 90 kDa, and also possesses 3′-5′ exonuclease proofreading function like other B-family DNAPs [53]. For the highest activity, Pwo DNAP needs a slightly more alkaline buffer condition, which may lead to the degradation of dNTPs, and thus dNTPs should be added right before the addition of Pwo DNAP when preparing the PCR solution [93]. Besides, the 3′-5′ exonuclease activity of this polymerase can lead to the degradation of primers and PCR products when the concentrations of dNTPs are low, and nuclease-resistant phosphorothionate protected primers can be used to solve this problem [10].

All the thermophilic DNAPs introduced above are from family A and B, as summarized in Table 1. The phylogenetic tree of these polymerases shown in Figure 1 exhibits their evolutionary relationships. Although some of these DNAPs are neither well known nor commercialized, their identifications have expanded the repertoire of thermophilic DNAPs, providing more candidates to be explored and engineered for various potential applications.

**Figure 2:**
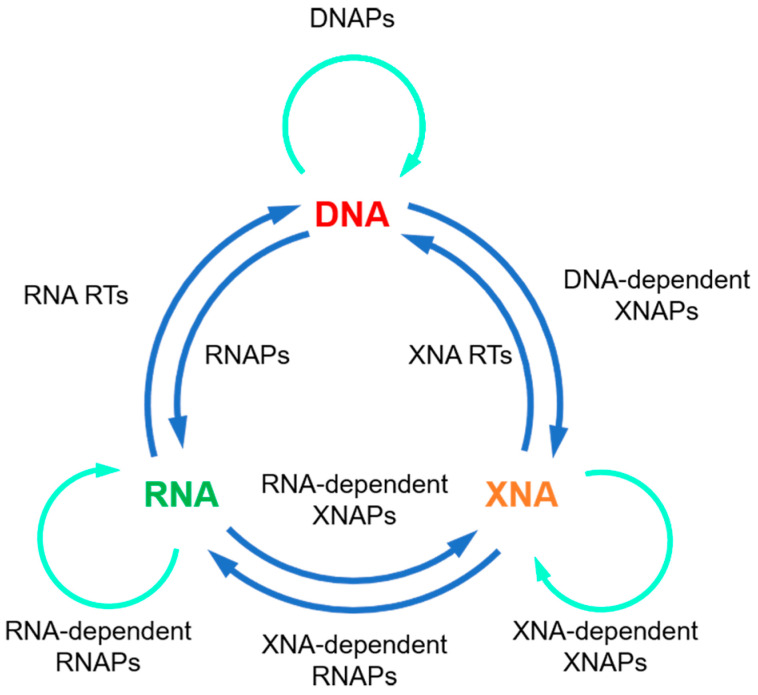
Expansion of the central dogma with XNAs and XNAPs. Green arrows: replication; blue arrows: transcription or reverse transcription.

Although natural thermophilic DNAPs are very efficient for DNA synthesis, and thus, have been broadly used in biotechnology, their activities of XNA synthesis are usually relatively poor, which severely limits their applications in xenobiology. In order to get efficient polymerases for the synthesis, reverse transcription, and even amplification or inter-transcription of XNAs (Figure 2), natural polymerases have to be engineered with various protein engineering strategies.

## 3. Strategies for Engineering Thermophilic Nucleic Acid Polymerases

The engineering of polymerases can be carried out via directed evolution, rational design, or semi-rational design [5,17,19,94]. Directed evolution mimics Darwinian evolution in nature, and yet with significantly shortened evolution time for desired phenotypic traits [95]. Random mutagenesis and/or recombination are carried out on the target polymerase genes with a much higher frequency than that of spontaneous mutagenesis or recombination in nature, followed by the selection or screening of desired mutants under artificial pressures. For polymerases with more structural information, rational or semi-rational approaches can be used to predict candidate residues or regions for mutagenesis, reducing the size of the polymerase library and the labor intensity for subsequent selection or screening [96].

### 3.1. Strategies for Mutant Generation or Library Construction

**Figure 3:**
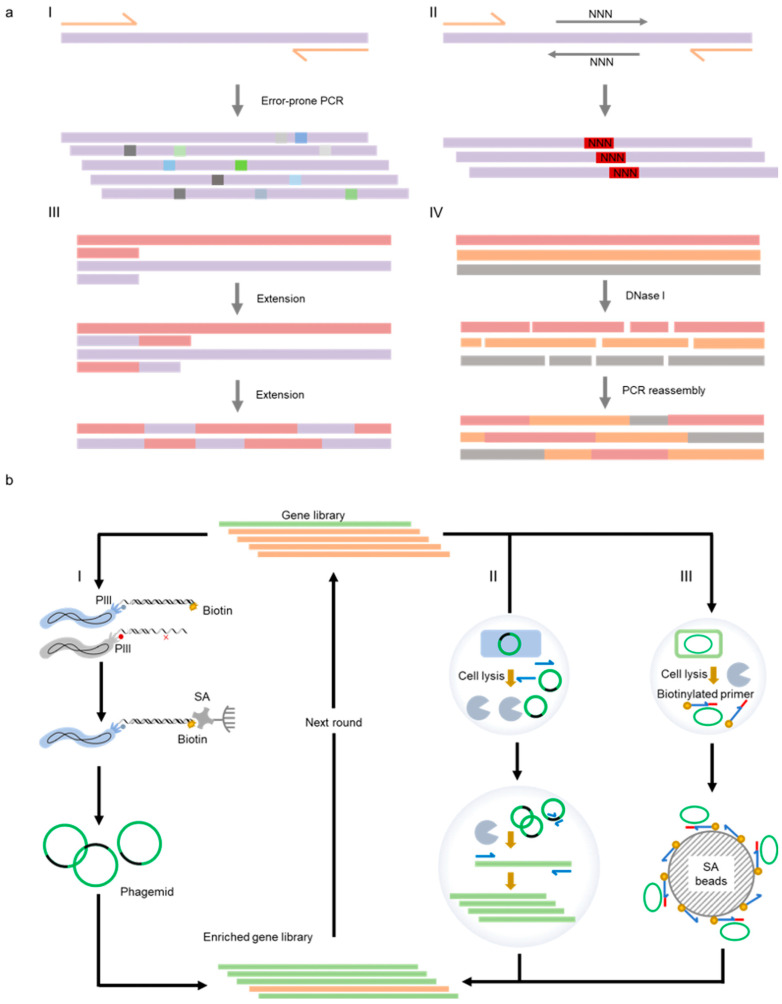
Representative methods employed for the generation of XNAPs. (**a**) Methods for the construction of polymerase libraries. I: error-prone PCR; II: site-directed saturation mutagenesis; III: gene shuffling by StEP; IV: gene shuffling by DNase I digestion and PCR reassembly; (**b**) methods for the selection of polymerase mutants. I: phage display; II: CSR; III: CST.

Error-prone PCR and DNA shuffling are two methods that are most extensively employed to randomize the gene of a target protein (Figure 3). Error-prone PCR is derived from standard PCR reaction, and yet polymerases of low fidelity and altered reaction conditions, including unbalanced concentrations of dNTPs and the addition of manganese ion are applied to increase the mutation rate of the target gene during amplification [97]. DNA shuffling provides a method to recombine homologous gene sequences, which is similar to natural homologous recombination but much more efficient [98]. Many strategies have been developed for DNA shuffling, including DNase I fragmentation and reassembly, staggered extension process (StEP), and synthetic shuffling. Traditional DNA shuffling involves DNase I digestion of a pool of homologous genes and subsequent reassembly of fragments by PCR [99]. Instead of random fragmentation and assembly, StEP uses the target genes as templates to create a recombinant library through multiple rounds of shortened polymerase-catalyzed extension [100]. In some cases, the addition of specific synthetic oligonucleotides during DNA shuffling can make the libraries more directional for studying the function of interest [101]. DNA shuffling usually requires high-sequence homology. For parental genes with insufficient homology, it may be a feasible method to optimize the shuffling template sequences through computer programs to improve the homology [102,103].

Rapid developments in sequencing, structure determination, and computational tools pave the way to the rational design of proteins. Through collection and analysis of existing sequence/structure-function data, candidate mutations of a protein for desired properties can be predicted. Site-directed mutagenesis is then carried out to generate target mutants or focused libraries. With a deeper understanding of sequence–structure-function relationships, even de novo design of proteins can be accomplished [104,105]. In past decades, many algorithms have been developed to facilitate structure prediction, design, and engineering of proteins, such as FoldX [106], Rosetta [107], I-Mutant [108], FRESCO [109], PROSS [110], and UniRep [111]. As an example of applying rational protein engineering approaches on polymerases, four Bst DNAP variants with enhanced thermostability have recently been obtained through MutCompute, an unsupervised machine learning algorithm [112].

Although having dramatically diminished the time and labor involved in the selection or screening of protein mutants, rational design requires extensive and in-depth data of sequence/structure-function relationships to improve the accuracy, which is not available for many proteins [113]. Combining the advantages of both directed evolution and rational design, semi-rational design has proven to be an effective tool for protein engineering. A small number of promising residues are identified based on computational simulation and analysis, leading to the construction of smaller but high-quality libraries and more efficient evolution processes. Various semi-rational approaches for protein engineering have been developed, including structure-based combinatorial protein engineering (SCOPE) [114], combinatorial active-site saturation test (CAST) [115], iterative saturation mutation (ISM) [116], sequence saturation mutagenesis (SeSaM) [117], protein sequence activity relationship algorithm (ProSAR) [118], and reconstructing evolutionary adaptive paths (REAP) [119]. Some of these approaches have been successfully used for the engineering of many thermophilic DNAPs, such as Bst DNAP and Taq DNAP [112,120].

### 3.2. Strategies for the Selection or Screening of Polymerase Libraries

Directed evolution is a powerful tool in the development of polymerases, in which the critical step is to build a high-throughput selection or screening method for the enrichment of active mutants. Selection or screening strategies for protein mutants are usually designed based on the binding of the proteins and their ligands, visualization of the catalytic activities of the enzymes, selective amplification of the target genes, or viability of the organisms [121]. The core for the selection or screening of a library is to connect the phenotypes of the mutants with their genotypes. At present, broadly used methods for selecting or screening polymerase mutants with unnatural activities mainly include methods based on the phage system, in vitro compartmentalization system, and multi-well plate system [17].

Based on the phage display technique, Romesberg and co-workers developed a polymerase selection system [120]. In this system, the polymerase library was co-displayed with the primer/template substrate on M13 phage particles, and successful extension of the primer with unnatural nucleoside triphosphates led to biotin labeling of the 3′-end of the primer, allowing the separation of active polymerase mutants from the library with streptavidin-coated beads. In another example, Liu and co-workers designed a phage-assisted continuous evolution (PACE) system, which could be used for iterative rounds of protein evolution without human intervention continuously [122]. This system correlated the desired activity of the target protein with the infectivity of the M13 phage and, thus, realized the rapid evolution of the protein along with the phage propagation.

In vitro compartmentalization is another strategy to build a linkage between genotypes and their corresponding phenotypes and has been broadly used in protein evolution. Some two decades ago, Tawfik and Griffiths developed “man-made cell-like compartments” using water-in-oil emulsions to generate separated micro-reactors, allowing the isolation of independent reactions and selection of promising protein variants [123]. Since then, emulsion-based compartmentalization has also been extensively applied to build polymerase evolution systems. Holliger and co-workers designed the compartmentalized self-replication (CSR) method based on microemulsion, in which polymerase variants were individually packaged into compartments of water-in-oil emulsion, together with PCR primers and nucleoside triphosphate substrates [124]. In this way, the genes of active variants were replicated by the polymerases that they encoded during thermocycling and enriched in the gene pool. Later, various derivative methods of CSR have been developed, including short-patch compartmentalized self-replication (spCSR) [125], reverse transcription-compartmentalized self-replication (RT-CSR) [126,127], compartmentalized partnered replication (CPR) [128], and high-temperature isothermal compartmentalized self-replication (HTI-CSR) [103].

To directly select or screen for hard-to-evolve polymerase mutants with XNA synthesis or reverse transcription activities, several other in vitro compartmentalization-based selection or screening strategies that do not rely on self-replication of the polymerase gene have been developed. For example, the compartmentalized self-tagging (CST) method was designed to select polymerases capable of XNA synthesis [129]. Similar to CSR, the polymerase pool was also compartmentalized with primers and nucleoside triphosphate substrates in water-in-oil emulsions to ensure the separation of individual variants and genotype–phenotype association. Different from CSR, CST was based on the tagging of a polymerase-encoding plasmid by extension of a short biotinylated primer when the polymerase had desired activity. Subsequent bead separation of the tagged plasmid allowed the enrichment of active polymerase variants. Later, to select for XNA RTs, Holliger and co-workers developed compartmentalized bead labeling (CBL), which relied on bead co-immobilization of the polymerase-encoding plasmids and primer/template complex for reverse transcription and subsequent fluorescent screening of the beads harboring desired variants [130].

To achieve a more controllable in vitro compartmentalization, microfluidic systems can be used to generate predefined compartments [131]. Recently, the Chaput group developed droplet-based optical polymerase sorting (DrOPS) method relying on microfluidic technology and cell sorting, in which polymerase variants were encapsulated with optical sensors for monitoring polymerase activity [132]. Successful extension of the primer by polymerase mutants led to the generation of fluorescence, and then, the water-in-oil-in-water or water-in-oil droplets were sorted by fluorescence-activated cell sorting (FACS) or fluorescence-activated droplet sorting (FADS) [133,134].

Although multi-well plate screening methods do not have throughputs as high as those of methods introduced above, they are still broadly used in the identification of polymerase variants from focused libraries with smaller size or from libraries pre-enriched with the methods introduced above [120,125,129,130]. In a typical multi-well plate screening method for polymerase mutants, the polymerase-mediated primer extension is correlated with the generation of colored or fluorescent products from enzymatic reactions, which can be directly monitored with a plate reader.

## 4. Thermophilic XNAPs

Thermophilic nucleic acid polymerases and their mutants have been extensively explored and used in the synthesis, reverse transcription, and even amplification of XNAs [5,135]. Although some other polymerases that are not high temperature tolerant, such as mutants of T7 RNAP, have also been used for the synthesis of modified nucleic acids [136], thermophilic nucleic acid polymerases are indispensable for XNA synthesis, reverse transcription, or amplification at high temperatures or with thermocycling programs, which are essential when tough templates with complex secondary structures are used or are important for higher yields.

Some thermophilic DNAPs, such as Taq, KlenTaq, Tth, KOD (exo^–^), KOD Dash, Vent (exo^–^), Pwo, Pfu, and Tgo DNAPs, demonstrate good tolerance to modifications on nucleobases [137,138,139,140,141,142] but are less tolerant to sugar modifications. However, syntheses or reverse transcriptions of different sugar-modified XNAs with limited lengths by certain polymerases have been reported, although not very efficient. For example, Taq DNAP was reported to be capable of reverse transcription or replication of hexose nucleic acid (HNA) with the length of a few nucleotides [143]. Bst DNAP has proven capable of reverse transcribing 2′-fluoro-arabino nucleic acid (FANA), α-L-threofuranosyl nucleic acid (TNA), and glycerol nucleic acid (GNA) [144,145]. Transcription or replication of short stretches of cyclohexenyl nucleic acid (CeNA) was demonstrated with Vent (exo^–^) DNAP, and reverse transcription of short CeNA was realized with Taq DNAP or Vent (exo^–^) DNAP [146]. Deep Vent (exo^–^) is able to reverse transcribe short stretches of TNA templates into DNA, and effectively incorporate all four 2′-deoxy-2′-fluoro-β-D-arabinonucleoside 5′-triphosphates (2′-F-araNTPs) on a DNA template to yield full-length FANA products [147,148]. In general, most natural polymerases show relatively narrow substrate specificities and limited activities towards XNAs. This is likely due to the fact that, in nature, to play their respective roles, polymerases have to possess stringent substrate specificities to accurately discriminate the sugars (deoxyribose and ribose) in their substrates, so that they can use the correct template (DNA or RNA) and the correct nucleoside triphosphates (dNTPs or NTPs) for the synthesis of their target products [149]. The introduction of unnatural sugars into the nucleic acids also usually leads to a great change in their structures, which may contribute to their difficult recognition by the natural polymerases as well [150]. To overcome the stringent substrate specificity and increase the activity towards unnatural substrates, natural polymerases have to be engineered.

Some commercial polymerase mutants demonstrate enhanced synthesis efficiency for modified nucleic acids. For example, Therminator DNAP, a variant of 9°N (exo^–^) DNAP, can incorporate various modified nucleotides [151,152,153] and even efficiently and faithfully synthesize long TNA oligonucleotides from DNA templates [153]. However, to realize efficient synthesis or reverse transcription of most of the fully substituted XNAs, the polymerases have to be further engineered via the directed evolution, rational design, or semi-rational design approaches summarized above.

Among thermophilic family A DNAPs, Taq DNAP and its truncated mutants, including SF and KlenTaq, are the most explored and engineered ones for expanded substrate repertoires. With a phage-display-based polymerase selection system, Romesberg and co-workers evolved an SF mutant, SFM19, that can incorporate 2′-O-methyl ribonucleoside triphosphates (2′-OMe-NTPs) on a DNA template [154]. Later, they further optimized the selection method and used SFM19 as the evolutionary starting point to evolve a series of polymerases, including SFM4-3, SFM4-6, and SFM4-9, that could transcribe or reverse transcribe fully 2′-OMe-modified oligonucleotides, or even PCR amplify partially 2′-OMe- or 2′-F-modified DNAs [120]. Further investigation demonstrated that these mutants could also synthesize or amplify other sugar-modified nucleic acids, including 2′-chloro (2′-Cl), 2′-amino (2′-Am), 2′-azido (2′-Az), and arabino-modified DNAs, and 2′-OMe- and 2′-F-modified RNAs [21,155,156]. Holliger and co-workers developed spCSR to select for variants of Taq DNAP, and obtained a mutant, AA40, with the ability to incorporate NTPs and sugar-modified nucleoside triphosphates [125].

Several thermophilic family B DNAPs have also been extensively engineered for the efficient synthesis and reverse transcription of various XNAs. For example, Holliger and co-workers used the CST method to select the libraries of TgoT DNAP (a Tgo DNAP mutant containing mutations V93Q, D141A, E143A, and A485L), and a mutant with HNA polymerase activity, Pol6G12, was obtained [129]. They also combined statistical correlation analysis (SCA) with activity screening or CST to develop a series of polymerases capable of synthesizing or reverse transcribing other XNAs, among which PolC7 can efficiently synthesize CeNA and LNA, PolD4K can efficiently synthesize ANA and FANA, RT521 can efficiently synthesize TNA and reverse-transcribe TNA, ANA, and FANA into DNA, while RT521K has good reverse transcription activity for CeNA and LNA. Later, by randomizing the positively charged and bulky residues of mutant RT521, which might lead to a steric clash between the polymerase surface and the P-ethyl-modification on the phosphate backbone, screening the libraries, and performing further site-directed mutagenesis, they successfully obtained mutant PGV2, which demonstrated substantially improved synthesis activity for a newly developed XNA with an uncharged backbone, alkyl phosphonate nucleic acids (phNA) [157]. Using CBL selection and plate-based screening methods, they further evolved a series of XNA RTs from mutant RT521K [130]. Among them, RT-TKK can efficiently reverse-transcribe D-altritol nucleic acid (AtNA). RT-C8, which was then evolved from RT-TKK, can efficiently reverse-transcribe 2′-OMe-RNA, and also has some extent of reverse transcription activity for P-α-S-phosphorothioate 2′-methoxyethyl RNA (PS 2′-MOE-RNA). Another derivative of RT-TKK, RT-H4, can reverse transcribe HNA much more efficiently than RT521K and RT-TKK.

Chaput and co-workers identified specificity-determining residues (SDRs) of the polymerase by analyzing the polymerase/DNA complex structure and screened for the beneficial mutations at SDR positions in a model polymerase scaffold [158]. By transferring these mutations to homologous proteins, a series of mutants that demonstrated RNA and TNA synthesis activities were rapidly developed from several family B DNAPs, including 9°N, Tgo, KOD, and Deep Vent DNAPs. They also successfully selected a manganese-independent TNA polymerase, 9n-YRI, from a site-saturation mutagenesis library of 9°N DNAP with the DrOPS method that they developed [132]. They further combined FADS sorting with deep mutational scanning to provide an unbiased screening of all possible single-point mutations in the finger subdomain of KOD (exo^–^) DNAP [134]. By screening mutants containing combinations of selected mutations, a double mutant, KOD-RS, which can conduct efficient TNA synthesis, was obtained, suggesting that polymerase specificity may be controlled by a small number of highly specific residues and more attention should be paid to these sites when engineering polymerases for the synthesis of specific nucleic acids. They later developed a programmed allelic mutation (PAM) strategy, applied it with DrOPS sorting, and successfully evolved a mutant with enhanced efficiency and specificity for TNA synthesis, Kod-RSGA, from mutant Kod-RS [159]. Herdewijn and co-workers reported 3′-2′ phosphonomethylthreosyl nucleic acid (tPhoNA or PMT) as a novel genetic material, and carried out stepwise engineering of TgoT DNAP to produce a PMT polymerase [160]. By introducing mutations that are related to XNA synthesis activity and screening for mutations at key residues based on previously reported mutants, they successfully obtained mutant TgoT-EPFLH, which can efficiently synthesize PMT. They also demonstrated that PMT could be efficiently reverse transcribed into DNA by both TgoT mutant RT521 and KOD mutant K.RT521K. Based on structural analysis, Hoshino et al. developed variants of KOD DNAP for LNA synthesis and reverse transcription, among which KOD-DGLNK can efficiently synthesize LNA from DNA, and KOD-DLK can efficiently reverse transcribe LNA into DNA [161]. These two mutants also demonstrated transcription or reverse transcription activity for 2′-OMe-RNA, respectively. Recently, Chaput and co-workers systematically compared some of the representative XNAPs obtained in previous works introduced above and demonstrated their diversity in thermostability and activity, specificity, and fidelity for the synthesis or reverse transcription of different nucleic acids, including RNA, FANA, ANA, HNA, TNA, and PMT [162].

## 5. Key Mutations in Engineered XNAPs

**Figure 4:**
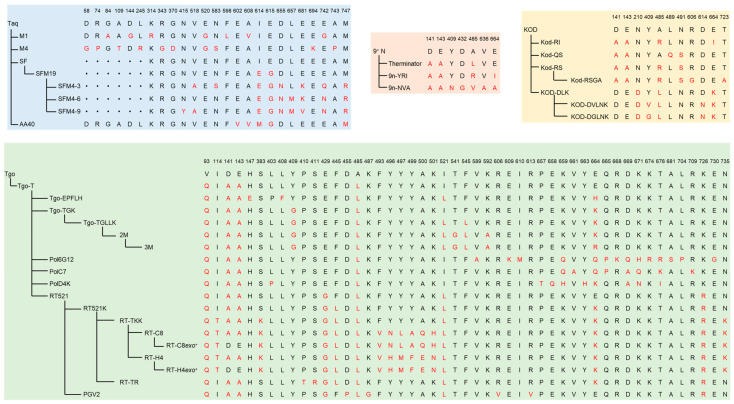
Summary of the relationships and mutations of some representative engineered XNAPs. The mutated amino acids in the engineered XNAPs are indicated in red.

**Figure 5:**
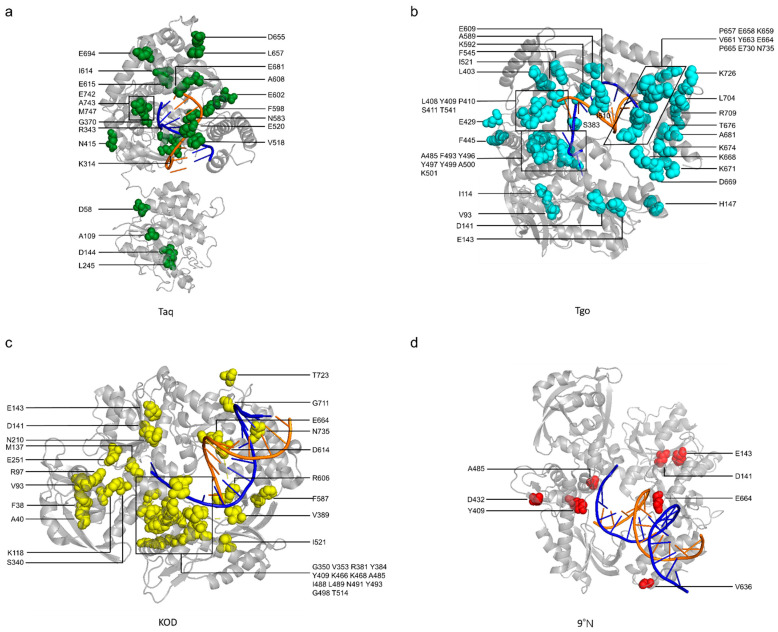
Distribution of the mutation sites in the engineered XNAPs. The mutation sites in engineered (**a**) Taq DNAP (green, PDB: 1TAU); (**b**) Tgo DNAP (cyan, PDB: 7B07); (**c**) KOD DNAP (yellow, PDB: 4K8Z); and (**d**) 9°N DNAP (red, PDB: 6ISF). The DNA templates and DNA primers are shown in blue and orange, respectively.

The mutations of representative engineered thermophilic XNAPs introduced above are summarized in Table 2 and Figure 4, and the distribution of the mutation sites in the structures of these engineered XNAPs is illustrated in Figure 5. Among these mutations, some are crucial for the gain or enhancement of the activities towards unnatural substrates, based on the analysis of polymerase structures and testing of the effects of specific mutations.

Steric gate residues are crucial for DNAPs to discriminate the deoxyribose of dNTPs from the ribose of NTPs, and usually need to be engineered to allow the polymerases to adopt sugar-modified substrates well [168]. As an example for thermophilic family A DNAPs, residue E615 is the steric gate residue of Taq DNAP, and the side chain of E615 packs on the 2′ position of the sugar ring directly, which consequently hinders the incorporation of nucleotides with a larger group at the 2′ position [125]. Therefore, E615 and its adjacent residue I614 are frequently mutated in Taq DNAP or SF mutants that can recognize sugar-modified nucleoside triphosphates [154]. In SF mutant SFM19 (I614E, E615G), which can incorporate 2′-OMe-modified nucleotides, E615 was mutated to Gly, which has a much smaller side chain and facilitates the access of 2′-OMe-NTPs. The same mutation was harbored by Taq DNAP mutant AA40 (E602V, A608V, I614M, E615G), which exhibited the activity of incorporating nucleotides with a few 2′-modifications, such as 2′-F, 2′-N_3_ and 2′-OMe [125]. SFM19 derivatives SFM4-3 (SFM19: V518A, N583S, D655N, E681K, E742Q, M747R), SFM4-6 (SFM19: D655N, L657M, E681K, E742N, M747R) and SFM4-9 (SFM19: N415Y, V518A, D655N, L657M, E681V, E742N, M747R) acquired more mutations beyond the steric gate residue, which further increased their processivity and allowed them to efficiently transcribe, reverse transcribe, or even amplify 2′-modified nucleic acids [16,120,156]. Holliger and co-workers obtained two mutants of Taq DNAPs, M1 (G84A, D144G, K314R, E520G, F598L, A608V, E742G) and M4 (D58G, R74P, A109T, L245R, R343G, G370D, E520G, N583S, E694K, A743P), both of which contained the mutation of E520 to a smaller residue Gly, and acquired the ability for processing a diverse range of non-canonical substrates [163]. In principle, to bestow the polymerases with good reverse transcription or replication activities for XNAs, it is helpful to engineer their interaction with the template strand. Residues E742 and M747 are involved in the interaction of Taq DNAP with the template strand, and their mutations have been found to contribute to the enhanced reverse transcription activity of several Taq DNAP mutants by the introduction of a salt bridge between the side chain of the new amino acid and the phosphodiester group in the template or an increase in the positive charge [169,170,171]. Therefore, mutations at these two residues in SF mutants are likely important for the efficient reverse transcription and amplification of 2′-modified nucleic acids.

For the extensively explored and engineered thermophilic family B DNAPs, including 9°N, Tgo, and KOD DNAPs, mutations at several key residues are frequently included in mutants that are efficient for the synthesis of various XNAs [158]. Mutations D141A and E143A lead to the inactivation of the exonuclease activity of these polymerases, preventing the removal of the incorporated XNA nucleotides and, thus, are usually introduced into these enzymes before further engineering for better XNA synthesis activity. Besides D141A and E143A, mutation N210D of KOD DNAP can also make it deficient in exonuclease activity [88]. Mutation V93Q significantly reduces the uracil stalling of these polymerases and has been included in many mutants for XNA synthesis and reverse transcription [172]. Residue A485 was mutated to Leu in Therminator DNAP, which is a mutant of 9°N DNAP [153]. Mutation of A485 may affect the pocket shape of the active site and the substrate recognition [173] and is included in many efficient mutants for XNA synthesis. Similar to family A DNAPs, steric gate residues also play important roles in substrate discrimination in these family B DNAPs. Y409 and E664 have been identified as the steric-gate residue and the second steric-gate residue, respectively, of Tgo DNAP, and mutations of these two residues are usually found to be crucial for the efficient synthesis of RNA and many kinds of XNAs [164]. Residue I521 is close to the catalytic residue D542 and the active site of Tgo DNAP, and has been mutated to different amino acids in many mutants that demonstrate XNA RT activity, as well as several mutants that have good synthesis activity for some XNAs [129].

For 9°N DNAP, mutations at residues A485, Y409, and E664 significantly alter its TNA synthesis activity. For example, Therminator DNAP, which harbors mutation A485L, possesses high activity for TNA synthesis [174], and mutants 9n-YRI (D141A, E143A, A485R, E664I) and 9n-NVA (D141A, E143A, Y409N, D432G, A485V, V636A, E664A) are both efficient Mn^2+^-independent TNA polymerase with greatly enhanced fidelity [132].

Starting from Tgo DNAP mutant TgoT, which harbors mutations V93Q, D141A, E143A, and A485L, various XNAPs have been produced. Mutant TGK (TgoT: Y409G, E664K) has proven capable of synthesizing RNA, as well as pseudouridine-, 5-methyl-C-, 2′-F-, and 2′-Az-modified RNAs [164]. Besides these activities, mutant TGK also has the ability to synthesize short stretches of FANA, ANA, HNA, and TNA [162]. Mutant TGLLK (TgoT: Y409G, I521L, F545L, E664K) has the ability to incorporate 3′-deoxy- or 3′-O-methyl-NTPs to produce nucleic acids with 2′-5′ linkages [165]. Very recently, mutant 2M (TGLLK: T541G, K592A) and 3M (TGLLK: T541G, K592A, K664R), which included more mutations at the nascent-strand steric gate residues in the TGLLK scaffold, were found to be able to efficiently synthesize long MOE-RNA and 2′-OMe-RNA up to 750 nt [166]. Mutant Pol6G12 (TgoT: V589A, E609K, I610M, K659Q, E664Q, Q665P, R668K, D669Q, K671H, K674R, T676R, A681S, L704P, E730G) has proven an efficient HNA polymerase [129], and can also synthesize FANA [162]. Mutant PolC7 (TgoT: K659Q, V661A, E664Q, Q665P, D669A, K671Q, T676K, R709K) can synthesize CeNA and LNA. Mutant PolD4K (TgoT: L403P, P657T, E658Q, K659H, Y663H, E664K, D669A, K671N, T676I) is capable of synthesizing ANA and FANA, and is also able to synthesize TNA, HNA, PMT, and RNA [129,162]. Obviously, mutation at secondary steric gate residue E664 is critical for the synthesis activities of different XNAs, and appears in all these mutants of Tgo DNAP. A series of TgoT mutants harboring mutations at key residue I521 and many other residues have been selected for efficient reverse transcription of different XNAs [129]. Mutant RT521 (TgoT: E429G, I521L, K726R) is able to reverse-transcribe HNA, ANA, FANA, and TNA. Mutant RT521K contains additional mutations F445L and E664K than RT521, and is efficient for the reverse transcription of LNA and CeNA [130]. A range of RTs for tougher XNAs is derived from RT521K by including even more mutations. For example, mutant RT-TKK (RT521K: I114T, S383K, N735K) can efficiently reverse transcribe 2′-OMe-RNA and AtNA. Both residues S383 and N735 are mutated to a positively charged lysine, which possibly enhances the electrostatic interaction between the polymerase and the non-cognate template. Mutation I114T is located in the uracil-binding pocket and also contributes to the enhanced reverse transcription activity of 2′-OMe-RNA. Mutant RT-C8 (RT-TKK: F493V, Y496N, Y497L, Y499A, A500Q, K501H), which harbors many more mutations than RT-TKK, is even more efficient for the reverse transcription of 2′-OMe-RNA, and can also reverse transcribe 2′-MOE RNA and PS 2′-MOE-RNA. Another derivative mutant of RT-TKK, RT-H4 (RT-TKK: F493V, Y496H, Y497M, Y499F, A500E, K501N), can reverse transcribe HNA much more efficiently than RT-TKK. Reverse mutagenesis of the exonuclease activity-eliminating mutations of RT-C8 and RT-H4 led to the production of mutants RT-C8*exo*^+^ (RT-C8: A141D, A143E) and RT-H4*exo*^+^ (RT-H4: A141D, A143E), which restore the 3′-5′ exonuclease activity, and are the first XNA RTs with proofreading activity. Introduction of mutations P410T and S411R, which are located in the nucleotide-binding pocket based on the structure of highly homologous KOD DNAP, into RT521K generates mutant RT-TR (RT521K: P410T, S411R), which presents significantly improved fidelity of reverse transcription.

As introduced above, mutant 9n-YRI contains mutations A485R and E664I, which promote TNA synthesis in the absence of Mn^2+^. Due to the 91% sequence similarity between KOD and 9°N DNAPs, identical mutations have been transferred to KOD DNAP to produce a novel TNA polymerase. The resulting mutant Kod-RI (D141A, E143A, A485R, E664I) was also found to be an efficient TNA polymerase [158]. Furthermore, mutant Kod-RS (D141A, E143A, A485R, N491S) and Kod-QS (D141A, E143A, L489Q, N491S) showed reduced ability for DNA synthesis and stronger specificity for TNA substrates [134]. Chaput and coworkers speculated that the coordination between mutations A485R and N491S allows the polymerase to adapt to the structural changes of the non-cognate TNA/DNA duplex and the incoming TNA substrate. Mutant Kod-RSGA (D141A, E143A, A485R, N491S, R606G, T723A) exhibited even higher specificity for TNA substrates compared with Kod-RS, which revealed that R606G and T723A double mutations are contributing to converting KOD DNAP into a TNA polymerase [159]. To evaluate the effects of different mutations at key residues on the LNA synthesis performance of KOD DNAP, Hoshino et al. compared the LNA synthesis activity and fidelity of KOD mutants DVL, DVLK, DGLK, DVLNK, and DGLNK [161]. Other than Therminator mutation A485L and steric gate mutations Y409V (or Y409G) and E664K, D614N, which is located in the thumb subdomain and was found to contribute to the efficient synthesis of LNA and improved fidelity of incorporating 2′-OMe-NTPs, was also included in mutants KOD DVLNK and KOD DGLNK. Mutant KOD DGLNK (N210D, Y409G, A485L, D614N, E664K) was shown to be able to synthesize one kb long LNA and also efficiently synthesize 2′-OMe-RNA. In addition, both KOD DGLNK and KOD DLK (N210D, A485L, E664K) exhibited LNA reverse transcription activity, indicating that only three substitutions in KOD DNAP are required to make this polymerase an LNA reverse transcriptase. Notably, residue Y412 of Vent DNAP is homologous to the steric gate residue, Y409, of KOD DNAP, and mutation Y412G also reduces the substrate specificity of Vent DNAP and promotes the incorporation of 2′-modified nucleotides [175].

## 6. Application of XNAs and Thermophilic XNAPs

**Figure 6:**
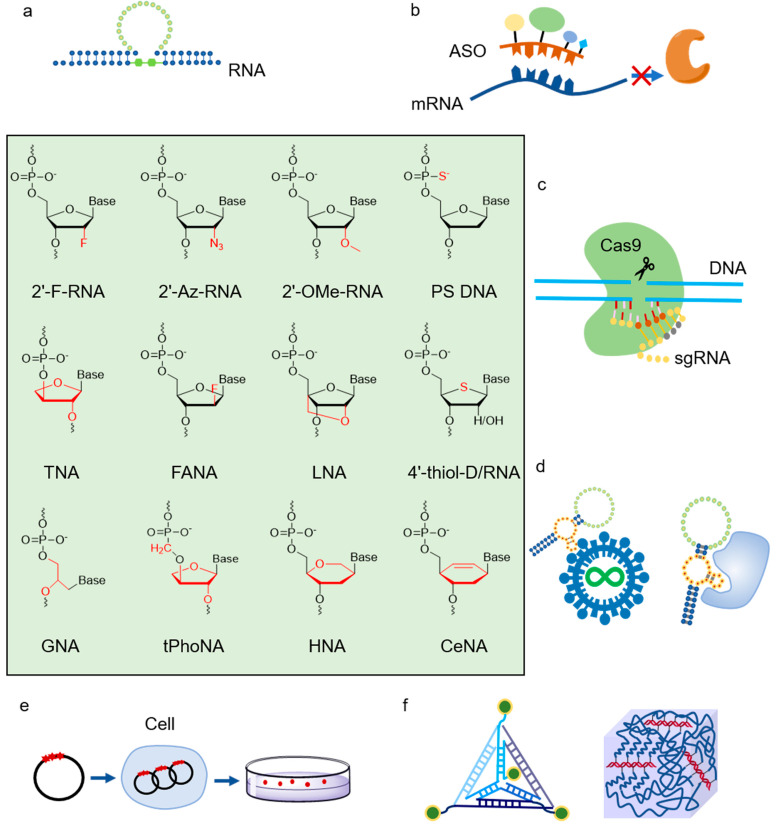
Application of XNAs and XNAPs. Modifications endow XNAs with expanded structural and functional diversities, and XNAPs further broaden the application scope of XNAs. (**a**) XNAzymes; (**b**) modified antisense oligonucleotides; (**c**) modified guide RNAs for CRISPR/Cas9 system; (**d**) XNA aptamers; (**e**) genetic information storage in living organisms; (**f**) XNA materials.

The unnatural moieties endow XNAs with expanded chemical and biological properties, which significantly broaden their application in various fields, spanning biotechnology, biomedicine, and nanotechnology [18] (Figure 6). The employment of efficient thermophilic XNAPs, either discovered or engineered, further facilitates the use of XNAs by allowing the transcription, reverse transcription, amplification, or evolution of them when needed [176].

Antisense oligonucleotides (ASOs) are short nucleic acids that can complementarily bind with target RNAs, such as mRNAs and miRNAs, to influence the expression of target genes through different mechanisms [177]. Development and exploration of chemically modified ASOs are urgently needed due to the limited properties of natural ASOs in nuclease resistance, target binding affinity, and pharmacokinetics. The first and the most broadly used chemical modification introduced into ASOs is phosphorothioate (PS), in which the non-bridging oxygen atom in the phosphodiester backbone is replaced by a sulfur atom [177,178]. The first ASO drug for treating cytomegalovirus retinitis approved by the US FDA, Vitravene (Fomivirsen), is PS modified [177,179]. However, the PS modification reduces its binding affinity to the target, resulting in an increased effective dose and accompanying toxicity [177,180,181,182]. Another ASO drug that has also received FDA approval is Kynamro (mipomersen sodium) [183]. It was modified with both PS and 2′-MOE to improve nuclease resistance and target RNA binding affinity [184]. In addition to 2′-MOE, FANA modification was also applied together with PS modification to produce ASOs with improved nuclease resistance and high binding affinity [185,186]. One of the most employed oligonucleotide modifications in recent years is LNA, which has a methylene bridge between the 2′-O and the 4′-C of the sugar ring [187]. It is characterized by a structure similar to RNA, high target binding affinity and specificity, high stability, and low toxicity, and thus excellent for ASO modification [188].

Ribozymes are catalytic RNA molecules that play important roles in many biochemical processes of living organisms, ranging from RNA splicing to protein synthesis [189]. Ribozymes and DNAzymes, which are catalytic DNA molecules, have found broad applications in biotechnology, biomedicine, biosensing, and biomaterials [190,191]. With the rise of XNAs, the possibility of creating XNAzymes as novel catalysts gradually attracted more and more attention [22]. Employing the XNAPs that they evolved from TgoT DNAP, Holliger and co-workers developed ANA, FANA, HNA, and CeNA XNAzymes with RNA endonuclease, RNA ligase, or XNA ligase activities [192]. Later, Chaput and co-workers evolved a FANAzyme that had a much higher catalytic rate for RNA cleavage, with different versions of Bst DNAP that can efficiently transcribe and reverse transcribe FANA [193]. By modifying classic DNAzyme 10-23 with FANA and TNA nucleotides, they also developed XNAzyme X10-23, which demonstrated good RNA cleavage activity and enhanced nuclease resistance while eliminating product inhibition [194]. In X10-23, the nucleotides in the substrate binding arms were fully substituted with FANA nucleotides, which in principle, would lead to higher substrate binding affinity due to the higher stability of FANA/RNA heteroduplexes compared with DNA/RNA heteroduplexes [195]. In addition, residues G2 and T8 in the catalytic core of X10-23 were also substituted with FANA nucleotides since it was found that these substitutions synergistically led to a 50% increase in the activity compared with the parental enzyme. Meanwhile, both the 5′ and 3′ ends of X10-23 were modified with a TNA nucleotide to protect it from nuclease degradation. With X10-23, they successfully silenced green fluorescent protein (GFP) and endogenous Kirsten rat sarcoma viral oncogene (KRAS) persistently. Recently, Yu and co-workers reported a TNA enzyme T8-6 that can catalyze the formation of 2′-5′ phosphate bonds in RNA ligation reactions, which was selected using Kod-RI DNAP [196]. In addition, they also developed a TNA enzyme Tz1, capable of cleaving RNA substrates, and successfully achieved target gene silencing in vivo [197]. The selection in vitro for this TNA enzyme was performed with Kod-RI DNAP and Bst 2.0 DNAP.

Clustered, regularly interspaced short palindromic repeats (CRISPR)/Cas9 system is a powerful tool for gene editing that has been extensively applied in biotechnology [198]. Recently, the introduction of unnatural modifications into guide RNAs (gRNAs) was found to be useful for improving the performance of CRISPR/Cas9 system. For example, Cleveland and co-workers reported a chemically modified CRISPR RNA (crRNA) with PS, 2′-F, 2′-OMe, and S-constrained ethyl (cEt) modifications, which demonstrated enhanced biostability and binding affinity to transactivating CRISPR RNA (tracrRNA), and improved activity in gene editing [199]. Ryan et al. suggested a method that greatly improves the specificity and reduces the off-target effect of the CRISPR/Cas9 system, in which 2′-O-methyl-3′-phosphonoacetate modifications were introduced into guide RNAs (gRNAs) at specific sites [200]. Sontheimer and co-workers introduced a set of crRNAs and tracrRNAs heavily or fully modified with 2′-F, 2′-OMe, or PS, which demonstrated good gene editing activity [201]. Another approach to improve the specificity of the CRISPR/Cas9 system via chemical modification of crRNA was proposed by Hubbard and co-workers [202]. They introduced bridged nucleic acids (2′,4′-BNA^NC^ [N-Me]) and LNA into specific sites of crRNAs to disrupt the binding of crRNAs to the off-target sequences, thus greatly improving the gene editing precision of CRISPR/Cas9 system.

Aptamers are single-stranded nucleic acid molecules that can bind specifically to target molecules and are usually selected from an oligonucleotide library through a process called systematic evolution of ligands by exponential enrichment (SELEX) [203]. Due to the limited biological stability and binding affinity of natural aptamers, many chemically modified aptamers have been developed. The first aptamer drug approved by FDA, Macugen (Pegaptanib sodium), which targets vascular endothelial growth factor (VEGF), is modified with 2′-F and 2′-OMe [204]. Using XNAPs to transcribe, reverse transcribe, or amplify XNA libraries, XNA aptamers can be directly evolved via SELEX. For example, Romesberg and co-workers evolved fully 2′-OMe-modified or partially 2′-F-modified aptamers targeting human neutrophil elastase (HNE) with SF mutants that they evolved [16,205]. Heemstra and co-workers evolved the first TNA aptamer targeting a small molecule, ochratoxin A (OTA), using KOD RI TNA polymerase and a large fragment of Bst DNAP I to transcribe and reverse transcribe the TNA library, and demonstrated its significant biostability and high specificity to the target [206]. Highly specific HIV-1 integrase-binding FANA aptamers with dissociation constants reaching picomolar levels have also been obtained via SELEX, in which the FANA library was created with mutant D4K of Tgo DNAP [207,208].

To use XNAs as materials for genetic information storage, propagation, and retrieval has always been a key focus of xenobiology, which is challenging, especially when implemented in vivo. As one example of the initial efforts, Matsuda and co-workers reported that DNA templates containing 4′-thio-dT and 4′-thio-dC modifications, which were prepared by PCR amplification with KOD dash DNAP, can be transcribed into functional RNAs in mammalian cells [209]. Pezo et al. demonstrated that DNA templates containing a few CeNA, AraNA, or HNA nucleotides could be recognized by DNAP in *E. coli*, achieving in vivo transfer of genetic information from these XNAs to DNA [210]. When a few nucleotides around the cysteine 146 codon of the *thyA* gene were replaced by CeNA, AraNA, or HNA, the *thyA*-gene-encoded thymidylate synthase was still functional in *E. coli*. In another work, PN-DNA, containing P3′-N5′ phosphoramidate bonds, was developed by Herdewijn and co-workers, and its building block, 5′-amino-2′,5′-deoxycytidine 5′-N-triphosphate (NH-dCTP), was successfully incorporated into the R67DHFR gene, which encodes the trimethoprim resistance, using Klenow fragment or Vent (exo^–^) DNAP [211]. Through resistance screening, antibiotic-resistant colonies were obtained, implying PN-DNA is able to store genetic information in living cells. Another example of in vivo replication of chemically modified DNA comes from Tavassoli and colleagues, who reported successful expression of a green fluorescent protein gene, *iLOV*, that was constructed by click-linking oligonucleotides via 5′-azides and 3′-alkynes in *E. coli* [212].

In addition to the applications described above, XNAs are also widely used in biomaterials, DNA nanotechnology, biosensing, and other fields [18,156,213,214,215,216,217]. For example, Romesberg and co-workers used SFM4-3 polymerase to PCR amplify DNA containing 2′-Az-A for preparing novel hydrogels [154]. Holliger and co-workers employed their evolved XNAPs to synthesize four different XNA strands and assembled them into XNA nanostructures [218]. With the continuous development of novel XNAs and their polymerases, XNAs will find ever-increasing applications, resulting in further expansion of the scope and increase of the importance of xenobiology.

## 7. Conclusions and Perspective

Since their isolation from organisms thriving at high temperatures, thermophilic nucleic acid polymerases have found broad application in almost all areas of biology and biotechnology [11]. The use of these enzymes is the core of many fundamental enabling techniques, including PCR and DNA sequencing [1,219]. Most of these enzymes that have been extensively explored and applied belong to family A and B of DNAPs, and their properties and activities varied significantly with their sources, structures, and functions in their original hosts, which leads to their diverse application scopes [219].

The development of XNAs to expand the scope of genetic materials is one of the core aspects of xenobiology. XNAs are derived from DNA and RNA, but display greater diversity in properties and functions, due to the unnatural moieties introduced into their structural units [220,221]. Polymerases that can efficiently transcribe, reverse transcribe, or amplify XNAs are essential for the full play of XNA functions. Despite their broad application, most of the natural nucleic acid polymerases demonstrate relatively poor activity towards unnatural substrates, which impedes their direct use as XNAPs. While a few thermophilic DNAPs were found to have polymerase activities for limited kinds of XNAs, many more efficient polymerases for various XNAs have been produced by engineering several well-studied thermophilic DNAPs with the approaches of directed evolution, rational design, or semi-rational design [18,96,150]. The discovery of these XNAPs immediately led to many applications of XNAs, such as the production of stable aptamers and XNAzymes [16,222].

Although current XNAPs already possess unnatural activities that allow some applications of XNAs, more efforts are still needed to improve their properties and performances. For example, for many XNA polymerases, the XNA synthesis or reverse transcription efficiencies and fidelities are still not satisfactory, and the lengths of the XNA products are still limited. Polymerases that can directly replicate and even PCR amplify fully modified XNAs have to be developed for better and broader use of XNAs in the future. Undoubtedly, the rapid development of novel protein design and engineering strategies, exemplified by machine learning approaches, will significantly facilitate our efforts on further engineering existing XNAPs to be better ones and, thus, broaden the application of XNAs [113]. Furthermore, the efforts on developing XNAPs have so far been mainly focused on engineering family A and B DNAPs from thermophiles, and thermophilic polymerases of other families may also be explored and engineered for XNA synthesis, reverse transcription, and amplification activities in the future, which may provide us more and even better XNAPs for different application scenarios. Hopefully, XNAPs that perform as well as natural DNA and RNA polymerases can be produced in the future, which will make XNAs become genetic materials comparable to, and even much better in some aspects, than DNA and RNA, and contribute to bringing xenobiology into an unprecedentedly thriving era.

## Figures and Tables

**Table 1:** Representative thermophilic and hyperthermophilic DNAPs.

Family	DNAP	Source	Properties				Ref.
			5′-3′ Exo	3′-5′ Exo	Error Rate	Half-Life Time	
**A**	Taq	*Thermus aquaticus*	Yes	No	1.2 × 10^−5^–3.3 × 10^−6^	97.5 °C/9 min	[25,26]
	Tfi	*Thermus filiformis*	Yes	No	/	94 °C/40 min	[27]
	Tth	*Thermus thermophilus*	Yes	No	/	94 °C/20 min	[10,28]
	Tfl	*Thermus flavus*	Yes	No	/	95 °C/40 min	[9,29]
	Tca	*Thermus caldophilus*	Yes	No	/	95 °C/70 min	[30]
	TsK1	*Thermus scotoductus*	Yes	No	/	95 °C/15 min	[31]
	Bst	*Bacillus stearothermophilus*	Yes	No	/	/	[32]
	Bca	*Bacillus caldotenax*	Yes	No	/	/	[33]
	Bcav	*Bacillus caldovelox*	Yes	No	/	/	[34]
	Bsm	*Bacillus smithii*	Yes	No	/	/	[35]
	Gss	*Geobacillus* sp. 777	Yes	No	/	/	[36]
	Tma	*Thermotoga maritima*	Yes	Yes	/	/	[37]
	Tne	*Thermotoga neapolitana*	Yes	Yes	3.4 × 10^−5^	/	[38,39]
	Aae	*Aquifex aeolicus*	No	Yes	/	75 °C/6 h 85 °C/1.7 h	[40]
**B**	Tli	*Thermococcus litoralis*	No	Yes	2.8 × 10^−6^	100 °C/2 h	[41]
	KOD	*Thermococcus kodakaraensis*	No	Yes	2.6 × 10^−6^	95 °C/12 h	[42]
	9°N	*Thermococcus* sp. 9°N-7	No	Yes	/	/	[43]
	Tgo	*Thermococcus gorganarius*	No	Yes	3.3–2.2 × 10^−6^	/	[44]
	Tfu	*Thermococcus fumicolans*	No	Yes	5.3–0.9 × 10^−5^	100 °C/2 h	[45]
	TNA1	*Thermococcus* sp. NA1	No	Yes	2.2 × 10^−4^	95 °C/12.5 h 100 °C/3.5 h	[46]
	Tpe	*Thermococcus peptonophilus*	No	Yes	3.37 × 10^−6^	90 °C/4 h	[47]
	Tzi	*Thermococcus zilligii*	No	Yes	2 × 10^−6^	/	[48]
	Twa	*Thermococcus waiotapuensis*	No	Yes	7.4 × 10^−6^	99 °C/4 h	[49]
	Pfu	*Pyrococcus furiosus*	No	Yes	1.3 × 10^−6^	/	[50]
	Pst	*Pyrococcus* GB-D	No	Yes	2.7 × 10^−6^	95 °C/23 h	[50,51]
	Pab	*Pyrococcus abyssi*	No	Yes	0.66–1.39 × 10^−6^	100 °C/5 h	[52]
	Pwo	*Pyrococcus woesei*	No	Yes	/	95 °C/8 h	[10,53]

**Table 2:** Summary of engineered thermophilic and hyperthermophilic XNAPs.

Parental DNAP	Mutant	Method Employed for Engineering	Mutation Sites	Unnatural Activity	Ref.
**Taq**	AA40	spCSR	E602V, A608V, I614M, E615G	Synthesis of 2′-F, 2′-N_3_ and 2′-OMe-RNA	[125]
	SFM19	Phage display	I614E, E615G	Synthesis of 2′-OMe-modified RNA	[154]
	SFM4-3	Phage display	I614E, E615G, V518A, N583S, D655N, E681K, E742Q, M747R	Synthesis or amplification of 2′-OMe, 2′-F, 2′-Az, 2′-Cl, 2′-Am-modified DNA/RNA and ANA	[120]
	SFM4-6	Phage display	I614E, E615G, D655N, L657M, E681K, E742N, M747R	Synthesis of 2′-F-DNA and 2′-OMe-RNA	[120]
	SFM4-9	Phage display	I614E, E615G, N415Y, V518A, D655N, L657M, E681V, E742N, M747R	Synthesis of DNA from a 2′-F-DNA or 2′-OMe-RNA template	[120]
	M1	CSR	G84A, D144G, K314R, E520G, F598L, A608V, E742G	PCR of phosphorothioate or fluorescent dye-modified DNA	[163]
	M4	CSR	D58G, R74P, A109T, L245R, R343G, G370D, E520G, N583S, E694K, A743P	PCR of phosphorothioate or fluorescent dye-modified DNA	[163]
**Tgo**	Tgo-RI	SDM *	D141A, E143A, A485R, E664I	Synthesis of TNA	[158]
	Tgo TGK	SDM *	TgoT: Y409G, E664K	Synthesis of pseudouridine-, 5-methyl-C-, 2′-F-, 2′-Az-modified RNAs, FANA, ANA, HNA and TNA	[162,164]
	Tgo TGLLK	SDM *	TgoT: Y409G, I521L, F545L, E664K	Synthesis of 3′-deoxy- or 3′-O-methyl-modified RNA	[165]
	RT521	CST	TgoT: E429G, I521L, K726R	Synthesis of DNA from an HNA, ANA, FANA or tPhoNA template	[129]
	RT521K	CST	RT521: F445L, E664K	Synthesis of DNA from an LNA or CeNA template	[129]
	RT-TKK	CBL	RT521K: I114T, S383K, N735K	Synthesis of DNA from a 2′-OMe-RNA or AtNA template	[130]
	RT-C8	CBL	RT-TKK: F493V, Y496N, Y497L, Y499A, A500Q, K501H	Synthesis of DNA from a 2′-OMe-RNA, HNA, AtNA, 2′-MOE-RNA or PS 2′-MOE-RNA template	[130]
	RT-C8*exo*^+^	SDM *	RT-TKK: A141D, A143E, F493V, Y496N, Y497L, Y499A, A500Q, K501H	Synthesis of DNA from a 2′-OMe-RNA, HNA, AtNA, 2′-MOE-RNA or PS 2′-MOE-RNA template	[130]
	RT-H4	CBL	RT-TKK: F493V, Y496H, Y497M, Y499F, A500E, K501N	Synthesis of DNA from an HNA template	[130]
	RT-H4*exo^+^*	SDM *	RT-TKK: A141D, A143E, F493V, Y496H, Y497M, Y499F, A500E, K501N	Synthesis of DNA from an HNA template	[130]
	RT-TR	CBL	RT521K: P410T, S411R	Synthesis of DNA from a 2′-OMe-RNA, HNA, AtNAs, 2′-MOE-RNA or PS 2′-MOE-RNA template with enhanced fidelity	[130]
	PolC7	CST	TgoT: K659Q, V661A, E664Q, Q665P, D669A, K671Q, T676K, R709K	Synthesis of CeNA and LNA	[129]
	PolD4K	CST	TgoT: L403P, P657T, E658Q, K659H, Y663H, E664K, D669A, K671N, T676I	Synthesis of FANA, ANA, TNA, HNA, and PMT	[129,162]
	Pol6G12	CST	TgoT: V589A, E609K, I610M, K659Q, E664Q, Q665P, R668K, D669Q, K671H, K674R, T676R, A681S, L704P, E730G	Synthesis of HNA and FANA	[129,162]
	6G12-I521L	SDM *	Pol6G12: I521L	Synthesis of HNA and FANA	[162]
	Tgo EPFLH	SDM *	V93Q, D141A, E143A, H147E, L403P, L408F, A485L, I521L, E664H	Synthesis of PMT, ANA, TNA, FANA and tPhoNA	[160,162]
	2M	SDM *	TGLLK: T541G, K592A	Synthesis of 2′-MOE-RNA and 2′-OMe-RNA	[166]
	3M	SDM *	TGLLK: T541G, K592A, K664R	Synthesis of 2′-MOE-RNA and 2′-OMe-RNA	[166]
**KOD**	KOD DGLNK	SDM *	N210D, Y409G, A485L, D614N, E664K	Synthesis of 2′-OMe-RNA and LNA	[161]
	KOD DLK	SDM *	N210D, A485L, E664K	Synthesis of DNA from an LNA template	[161]
	Kod RI	SDM *	D141A, E143A, A485R, E664I	Synthesis of TNA	[158]
	Kod RS	DrOPS	D141A, E143A, A485R, N491S	Synthesis of TNA	[134]
	Kod QS	DrOPS	D141A, E143A, L489Q, N491S	Synthesis of TNA	[134]
	Kod RSGA	DrOPS	D141A, E143A, A485R, N491S, R606G, T723A	Synthesis of FANA, ANA, HNA, TNA, C5-modified TNA, and PMT	[159,162,167]
	KOD RTX	RT-CSR	F38L, R97M, K118I, M137L, R381H, Y384H, V389I, K466R, Y493L, T514I, I521L, F587L, E664K, G711V, N735K, W768R	Synthesis of DNA from a 2′-OMe-RNA template	[126]
	KOD RTX-Ome v6	RT-CSR	RTX: A40V, E251K, S340P, G350V, V353L, H381R, H384Y, K468N, I488L, G498A, K664R	Synthesis of DNA from a 2′-OMe-RNA template	[127]
	KOD RT521K	SDM *	V93E, D141A, E143A, A485L, I521L, E664K	Synthesis of DNA from a tPhoNA template	[160]
**9°N**	9°N Therminator	SDM *	D141A, E143A, A485L	Synthesis of TNA	[153]
	9n-YRI	DrOPS	D141A, E143A, A485R, E664I	Synthesis of TNA	[132]
	9n- NVA	DrOPS	D141A, E143A, V409N, A485V, E664A, D432G, V636A	Synthesis of TNA	[132]
**Deep Vent**	Deep Vent RI	SDM *	D141A, E143A, A485R, E664I	Synthesis of TNA	[158]

* SDM: site-directed mutagenesis (including site-directed saturation mutagenesis).
